# Experiences of patients at high risk of opioid overdose accessing emergency department and behavioral health interventions: a qualitative analysis in an urban emergency department

**DOI:** 10.1186/s12913-023-09387-7

**Published:** 2023-04-18

**Authors:** Alexandra B. Collins, Janette Baird, Evelyn Nimaja, Yokabed Ashenafi, Melissa A. Clark, Francesca L. Beaudoin

**Affiliations:** 1 Department of Epidemiology, Brown University School of Public Health, 121 S Main Street, Providence, RI USA; 2 Department of Emergency Medicine, Warrant Alpert Medical School of Brown University, 55 Claverick Street 2Nd Floor, Providence, RI 02903 USA; 3 Department of Health Services, Policy & Practice, Brown University School of Public Health, Providence, RI USA

**Keywords:** Overdose, Emergency department, Peer worker, Social worker, Harm reduction

## Abstract

**Background:**

Emergency Departments (EDs) have become critical ‘touchpoints’ for the identification and early engagement of patients at risk of overdose or who have an opioid use disorder (OUD). Our objectives were to examine patients’ ED experiences, identify barriers and facilitators of service uptake in ED settings, and explore patients’ experiences with ED staff.

**Methods:**

This qualitative study was part of a randomized controlled trial that evaluated the effectiveness of clinical social workers and certified peer recovery specialists in increasing treatment uptake and reducing opioid overdose rates for people with OUD. Between September 2019 and March 2020, semi-structured interviews were conducted 19 participants from the trial. Interviews sought to assess participants’ ED care experiences across intervention type (i.e., clinical social worker or peer recovery specialist). Participants were purposively sampled across intervention arm (social work, *n* = 11; peer recovery specialist, *n* = 7; control, *n* = 1). Data were analyzed thematically with a focus on participant experiences in the ED and social and structural factors shaping care experiences and service utilization.

**Results:**

Participants reported varied ED experiences, including instances of discrimination and stigma due to their substance use. However, participants underscored the need for increased engagement of people with lived experience in ED settings, including the use of peer recovery specialists. Participants highlighted that ED provider interactions were critical drivers of shaping care and service utilization and needed to be improved across EDs to improve post-overdose care.

**Conclusions:**

While the ED provides an opportunity to reach patients at risk of overdose, our results demonstrate how ED-based interactions and service provision can impact ED care engagement and service utilization. Modifications to care delivery may improve experiences for patients with OUD or at high risk for overdose.

**Trial registration:**

Clinical trial registration: NCT03684681.

## Background

The United States (US) is continuing to be impacted by a decades-long overdose crisis, with more than 100,000 individuals dying of a fatal drug overdose in 2021 alone [7, 35]. Emergency department (ED) visits following an opioid-related overdose have also risen dramatically across the US in the last decade [48]—including during the COVID-19 pandemic [47]—with engagement now exceeding 140,000 visits annually [17, 53]. Individuals who are admitted to the ED for an overdose face an increased risk of subsequent fatal and non-fatal overdoses [27, 39, 58] making EDs potentially critical settings to provide substance use-related treatment and services [20, 54].

ED-based efforts aimed at addressing subsequent overdose risk and expanding access to treatment and support services have included programs such as take-home naloxone distribution [14, 23, 26] and ED-based buprenorphine initiation [13, 18, 49]. More recently, there has also been a push to integrate people with lived substance use experience into ED staffing models (e.g., peer navigators, peer recovery specialists) to better support clinical care delivery, connections to treatment, and to address stigma faced by people who use drugs within ED settings [11, 45]. While peer roles vary (e.g., certified training program, less formal training), peer recovery specialists are individuals with lived experience who support clients in accessing clinical and ancillary care (e.g., medications for opioid us disorder, community-based resources) [3, 30].

Previous research has demonstrated the acceptability and feasibility of peer-led behavioral health interventions (e.g., counseling) in ED settings and the potential for such interventions to improve opioid use disorder (OUD) treatment uptake and limit re-hospitalization for overdose [30, 43, 44, 57]. Within acute care settings, peer recovery specialists have also been documented as serving a critical role for patients during and post-hospitalization, including improving engagement in care [28], as well as for clinical providers when working with patients who use drugs [10, 11]. However, there is a dearth of research examining patients’ perceptions and utilization of peer-led ED supports following an overdose and how these interactions shape ED experiences for people who use drugs.

Given that people who use drugs often face discrimination and stigma when accessing clinical care [8, 32, 33, 52], it is imperative to understand how the integration of peer recovery specialists may mitigate these barriers and improve their ED experience. This analysis examined the ED experiences of people at high risk of overdose who were assigned to either a licensed clinical social worker or certified peer recovery specialist as part of a larger clinical trial in Rhode Island [4]. In our study setting, peer recovery specialists are people with lived substance use experience who have been in recovery at least two years and have completed a 45-h training program and 500 h of supervised work experience [1, 57]. Under statewide policy [42], peer recovery specialists are one of a suite of services and resources (e.g., naloxone distribution, induction of medications for OUD) to be offered to patients presenting to any Rhode Island ED after an overdose or with an OUD [10, 43, 44]. Here, we aimed to understand participants’ perceptions of their ED clinical care experiences across provider and staff type, and to identify barriers and facilitators of resource utilization within ED settings.

## Methods

We draw upon semi-structured interviews conducted with people receiving ED-based care who were part of a larger randomized control trial. The larger trial compared the effectiveness of peer recovery specialists and ED-based social workers on treatment uptake, future opioid overdose rates, and ED utilization for opioid overdose in Rhode Island [16]. In brief, participants were randomly assigned to one of two study arms (peer recovery specialist or clinical social worker). A control arm had initially been implemented but was closed 6 months after the study launched due to futility [4]. Each intervention arm included a consultation in the ED that sought to address participants' immediate (e.g., MOUD access, naloxone) and broader social-structural (e.g., housing or transportation supports) needs. Interventions by social workers were delivered during a single interaction within the ED, whereas peer recovery specialists followed-up with participants for three months after their ED visit [4, 16]. All study activities were approved by the Institutional Review Board (IRB) at the primary study site, and all study methods were carried out in accordance with the IRB-approved study protocols and regulations. Participants were recruited from the parent study which took place in two EDs (level 1 and level 2 trauma centers). Full-time research assistants were available to recruit participants presenting in the ED (24 h per day, 7 days per week). Potential participants in the parent study were identified by the research assistants using electronic medical records and via referral from treating ED providers [16]. A research assistant then met with the potential participant, told them about the study, and if interested, screened them in the ED.

Between September 2019 and March 2020, semi-structured interviews were conducted with 19 participants from the parent trial to assess their ED care experiences across intervention type (i.e., clinical social worker or peer recovery specialist). Participants were eligible if they were: at least 18 years of age; able to conduct the study in English; and presenting for an opioid overdose in the ED or deemed to be at high risk for an opioid overdose. High overdose risk categorization included currently being treated in the ED for an opioid overdose; had an opioid overdose in the prior 12 months; were receiving treatment for opioid withdrawal; or were receiving treatment for an injection-related outcome (e.g., abscess, soft tissue injection) [4, 16]. The qualitative team was comprised of two research assistants and four members of the investigative team. The study team were highly trained with prior qualitative research experience and training. The research assistants received significant training in qualitative methods and research ethics by a senior member of the research team (JB) prior to data collection with weekly audio checks and team check-ins occurring throughout the study period to ensure rigor of data collection processes. All team members were women, and one was also a practicing clinician.

**Fig. 1:**
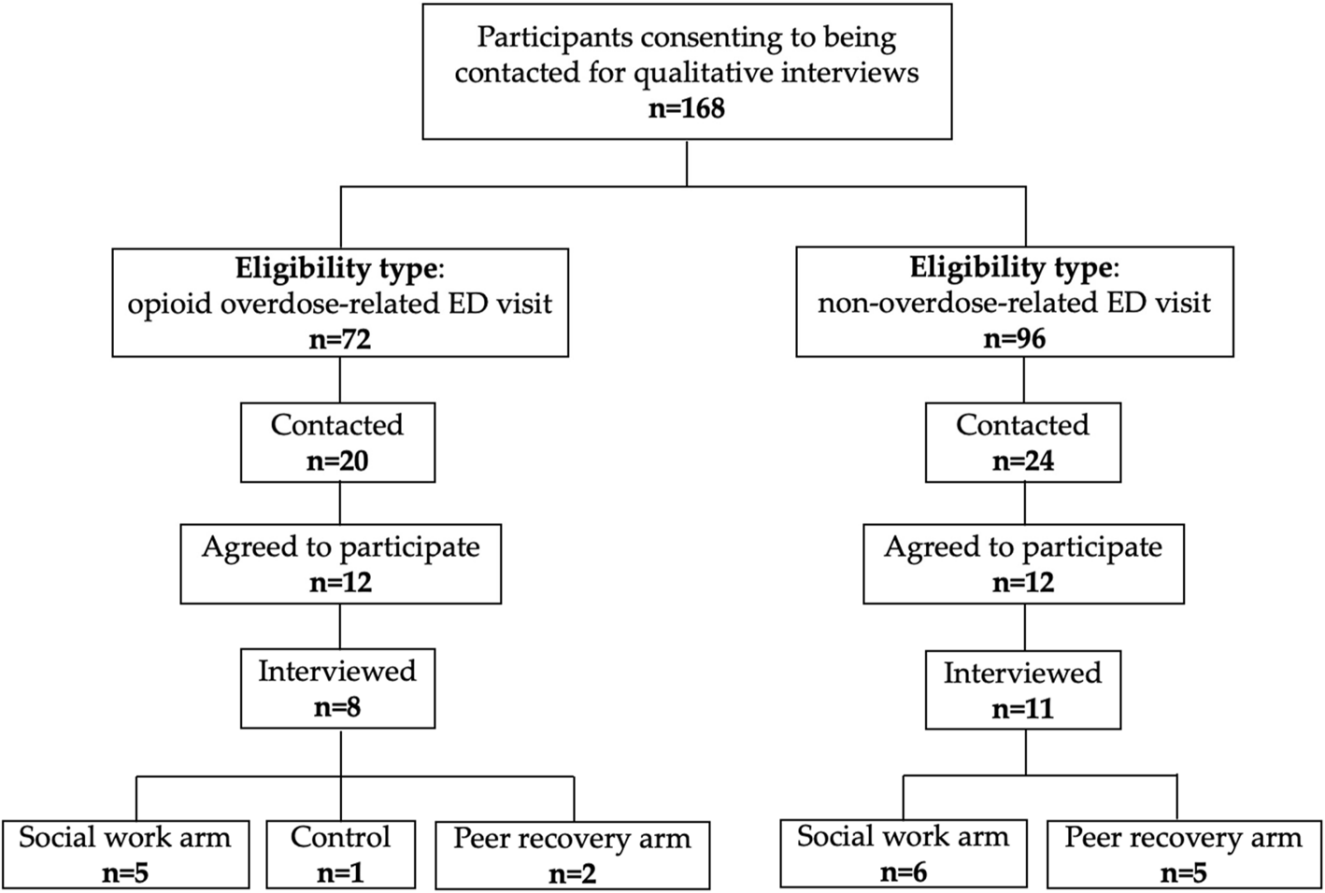
Participant recruitment

Participants from the parent study who had agreed to be contacted for a qualitative interview during the informed consent process (*n* = 168) were purposively sampled. We aimed to recruit a total of 30 participants stratified by reason for ED visit (overdose-related visit [*n* = 15] or non-overdose-related visit [*n* = 15]). Within each group, participants were purposively sampled to ensure representation across intervention arm (i.e., social worker, peer recovery specialists, control) (see Fig. 1). Because most ED visits for opioid overdose in our study setting are among white males aged 25–44, we also sought to have at least 25% of the study sample be female [41]. All interviews took place within 10 days of participants’ ED visit. Two women BA-level research assistants on the study team attempted to contact all trial participants who consented to being followed-up with about the qualitative sub-study (*n* = 168) by phone. Recruitment continued until representativeness across study arm and ED visit type were obtained. However, as the control arm of the trial was stopped after 6 months, sampling was modified to include 6–7 participants from each arm (social worker, peer navigator).

A total of 44 trial participants were reached, with the remainder unable to be contacted. The research assistant explained the qualitative sub-study and 24 participants agreed to participate (see Fig. 1). Interviews were scheduled by the research assistants. However, due to the COVID-19 pandemic and its impact on hospital settings, COVID-related policies (e.g., no in-person data collection activities), and participant follow-up interviews were stopped in March 2020 after completing 19 interviews. Participants interviewed were representative of the participant sample in the parent study [4] and included participants in the social work arm (*n* = 11), peer recovery specialist arm (*n* = 7), and control arm (*n* = 1).

Participants were interviewed in private rooms in the study site by one of two women research assistants who had been trained in qualitative research methods (EN, YA). Verbal informed consent was obtained prior to starting the interviews and interviewers briefly shared their interest in the study topic with participants. Interviews were facilitated using a semi-structured interview guide developed by members of the study team and informed by the study’s aims, relevant literature, and ED clinical expertise of two study team members. Topics included experiences within ED settings (e.g., discrimination, facilitators to care, services discussed with providers), experiences with the treatment arm randomized to (e.g., peer navigator, social worker) including pre- and post- ED visit, perceptions of behavioral counseling received, and ED-based harm reduction resources (e.g., naloxone, medications for OUD). Interviews ranged from 25 to 60 min, were audio-recorded, and transcribed verbatim by a professional transcription company. All transcripts were reviewed for accuracy by the study RAs. All participants received a $50 gift card honorarium. The study team met weekly to discuss and reflect on emerging findings during the study period, including data saturation.

Data were coded and analyzed thematically in NVivo12 using inductive and deductive approaches [6]. An initial coding framework was developed by the study team using a priori codes drawn from the interview guide (e.g., ED-based stigma, services offered in the ED, experience with medications for OUD) and informed by team discussions during data collection. The coding framework was regularly refined to capture additional codes that emerged through line-by-line coding [12]. Three study team members coded the first five transcripts to ensure codes were applied the same across transcripts and coders (EN, YA, JB). The remaining transcripts were then independently reviewed and coded by two team members (EN, YA). Differences in coding were reconciled by consensus among coders. Thematic analysis was guided by participant experiences in the ED, with a focus on social and structural factors shaping care experiences and service utilization. Feedback was solicited during the analysis process from study team members with ED-based medical expertise (FB) in addition to input from the broader team (ABC, JB, MAC).

## Results

The average age of participants was 44 years, and most participants were male (68%) and white (69%) (see Table 1). Among the 19 participants, eight presented to the ED after an opioid overdose, 11 were randomized to receive an intervention from a clinical social worker, seven from a peer recovery specialist, and one declined the behavioral intervention, but was enrolled in the parent study. While the length of time participants were in the ED at time of study enrollment varied, all had prior ED experiences, including at the study sites.

**Table 1:** Participant characteristics

Demographic characteristic	n (%)
**Sex**	
Female	6 (32%)
Male	13 (68%)
**Age (years)**	
Median (IQR)	44 (34, 50)
**Race**	
White	13 (69%)
Non-white	5 (26%)
Don’t know/refused	1 (5%)
**Ethnicity**	
Hispanic or Latino/a	3 (16%)
Non-Hispanic or Latino/a	15 (79%)
Don’t know/refused	1 (5%)
**Education**	
Trade or technical school	2 (10%)
Some high school	7 (37%)
Finished high school or GED	4 (21%)
Some college	3 (16%)
College or university degree or higher	3 (16%)
**Study arm assessment**	
Social worker	11 (58%)
Peer recovery specialist	7 (37%)
Refused to be randomized	1 (5%)
**Reason for ED visit**	
Opioid overdose	8 (42%)
Non-opioid overdose	11 (58%)

### “They do a good job of not making you feel uncomfortable”: positive ED interactions

Participants’ narratives underscored how their ED visits had been shaped by provider and staff interactions. For most participants, perceived attitudes of providers and hospital staff during care provision were a critical element to shaping their overall ED experience and willingness to seek care in the future. Several participants expressed that providers’ tone made them feel more comfortable, and how helpful it was to not be “*pushed*” into talking. One participant explained: “*They do a good job of not making you feel uncomfortable. Not asking too much. Not pushing you if you’re not into, you know, into talking*” (Participant 2). Not feeling pushed by providers to open up was further stressed as an important step in rapport building during ED encounters after an overdose. One participant explained how this can improve patients’ experiences:

*She* [the nurse] *was a hot sketch. She was funny, she was down to earth. You know, she talked on my level – she didn’t talk doctor. …She talked to me about everyday things not just experiences and what I was doing there. Just everyday things. And*, [she] *just felt like a friend, you know? Like a friend conversing with each other.* [Participant 5]

Taking time to ‘get to know’ patients was stressed by other participants as impacting their willingness to learn about other services. One participant stressed how providers should engage in meaningful communication before suggesting treatment options:

*Usually when people overdose, it’s either doctors or EMTs, they’re always suggesting detox. That’s like the first thing. At least get to know the person first before you ask them questions like that. Like talk to them for a second and then ask that question. Don’t come off like, ‘Are you going to a detox?’ That’s not going to make me want to get sober.* [Participant 1]

Patient-provider communication approaches that were ‘judgement-free’ were described as facilitating more open dialogue about participants’ circumstances and areas where health and ancillary support was needed. For example, one participant described their experiences when referred to a social worker in the ED:

*He asked me* [if I wanted one]*. He didn’t tell me. Like I was telling him* [doctor] *about how, you know, I keep relapsing. I don’t want to die and that I want to get clean. And he was like, ‘Ok well I’m going to send some people your way and I think that they’ll help you*. [Participant 4]

Here, the ability to talk openly with providers allowed for collaborative discussions about needed steps thereby reinforcing participants’ agency in the clinical setting.

### “Why do the job?”: Experiences of discrimination in the ED

While many participants described having positive ED interactions, others underscored how an OUD diagnosis also contributed to being judged during ED encounters. Several participants described being labeled as a “*drug abuser*” by hospital staff due to their OUD diagnosis, which resulted in their clinical needs being taken less seriously. For participants with co-occurring health conditions (e.g., chronic pain, blood disorders), providers’ assumptions based on their OUD diagnosis were described as “*detrimental*” and leading to barriers to care. One participant with sickle cell anemia explained:

*Some come in just for pain medication and they’re not in pain and they get addicted to it. So right there, any person with sickle-cell is being judged… I’m being judged. Even when I go on the floors, the nurses, if they don’t know me, I’m being judged. …So my whole experience down at the ED, it wasn’t all that great. First feeling embarrassed, then feeling uncomfortable because these nurses were judging me. I think they were judging me*. [Participant 6]

However, some participants simultaneously sought to reassert their agency in these situations by choosing—or not choosing—to share substance use-related information. For some participants, choosing to disclose their OUD diagnosis with providers prior to them seeing it in their charts was positioned as useful for shaping care:

*I just wanted to be forthcoming when I got to the ER to let them know that I am in recovery but I needed something for pain. And so, I had already put that on their radar before you know they had anyone look it up or whatever.* [Participant 15]

By being open about their OUD, participants sought to position their need for pain medication as legitimate thereby disrupting typical assumptions they had previously experienced. However, other participants chose to not fully disclose their health or social experiences to providers to reduce risk of discrimination. One participant described this approach, explaining:

*I really felt like I didn’t want to ask for help, or even disclose anything going on with me. Because, how can the hospital help you if you don’t tell them the truth? But, why do you want to tell them the truth if they’re going to treat you like that?* [Participant 8]

While participants acknowledged that withholding information from providers undermined having their medical needs met, it simultaneously allowed them to reassert their agency, thereby protecting themselves from potential discrimination.

Notably, there was an awareness across participants that not all people accessing ED services had the same experiences. One participant underscored these variable experiences, explaining:

*I was asked questions* [about drug use] *but they were more concerned with my fractures. Not that they weren’t concerned, but I think they kind of, I don’t know. …I don’t think that they thought or treated me different. And I mean, look at me, like I don’t have track marks. Like, I’m not a drug addict and I think they kind of got that it* [my overdose] *was a mistake*. [Participant 14]

For this participant, a lack of physical representations of substance use on their body (e.g., “*I don’t have track marks*”) was positioned as impacting the treatment they received in EDs and how they were ‘labeled’—or not—by providers. Conversely, several participants described how providers often made assumptions based on patient behaviors (e.g., napping) in addition to appearance. One participant shared:

*They kept thinking I was on something because I kept falling asleep. But I wasn’t even on anything, I just didn’t sleep the night before. I kept nodding off – I was up the night before cause my back was so messed up. …I just kept hearing them whispering and then they finally flat out asked me. And I’m like, ‘I’m fucking tired*.’ [Participant 11]

Additionally, participants described how the communication approaches of ED-based personnel (e.g., social workers, police, providers) resulted in them being “*talked down to*” or communicated with in ways that undermined feeling cared. One participant described:

*Talk to me like you want to be here. Don't talk to me like it's just another 12-hour shift. …Do your job and that's it...I'm telling you it was the worst experience ever. Honestly, I thought they felt like they were just wasting their time.* [Participant 18]

Like Participant 18, others who had negative experiences with ED-based providers stressed that lack of compassion led to “*wasting* [patients’] *time*” and questioned why certain behavioral and medical providers worked with people following an overdose: “*If you ain’t going to stay and listen* [to patients] *– why do the job? You’re wasting their time and yours*” [Participant 6].

### “They need someone who’s been in that position”: the importance of lived experience

Given the challenges participants described regarding ED communication following an overdose, many participants stressed the importance of having people with lived experience support them in ED settings. For many, “*see*[ing] *somebody who’s been through it and won a couple times*” was critical to improving their ED experiences and next steps. One participant described:

*They don’t need a doctor in their face saying they should have done this or ‘You need to do this.’ They need someone who’s been in that position and will listen to them. …Nobody wants to be told, ‘You shouldn’t have done this and this,* Miss [Name],’ *and ‘You’re killing yourself Miss [Name].’ I know all this. Seriously, I get it. Get somebody in my face who almost killed themselves that I can talk to and relate to. Nobody wants to be talked down to. It sucks.* [Participant 9]

Continuing, this participant shared how having peer recovery specialists “*who works close to a doctor and knows what it is to be in your position*” could improve post-overdose ED experiences for patients. Similar sentiments were shared across participants who described engaging with peer recovery specialists as being “*more comfortable than social workers*” due to their lived experience, explaining: “*I guess it’s easier to talk to someone that’s been through it better than someone who just went to school for it*” (Participant 4).

While lived experience was deemed critical to improving interactions and care pathways in the ED, several participants noted how social workers could “*pull more strings*” than peer recovery specialists thereby improving access to treatment and other resources. One participant explained:

*They’re* [social worker] *somebody that can help you get your shit together and, you know, guide you. Give you the resources you need to get whatever it is you need to get, whether it’s out of a bad home into a good home, whatever.* [Participant 5]

While social workers were viewed by some participants as affording patients with improved access to services and resources, many stressed how increased support with next steps in service engagement was necessary. One participant explained: “*It felt like, ‘All right, well here’s a list of papers and, you know, you go make the phone call’”* (Participant 3). Participants described how options for a more hands-on approach during referral processes was necessary to supporting patients, yet one that was often missing.

For participants, gaps in support during referrals often intersected with a lack of lived experience among providers who participants felt did not know the intricacies of substance use. One participant explained: “*I’d rather talk to somebody who’s been through it than somebody who doesn’t know anything about it except what they learned in school or whatever*” (Participant 12). Similarly, other participants acknowledged how dedicated trainings still fail to provide the medical and behavioral healthcare providers with knowledge that they felt was important when working with patients who use drugs:

*The only education I can think of is if they went through it. That’s the only education you can have is going through it. I mean you can’t learn this in school. Well, you can learn it in school, but to live it is something different*. …[So] *listen to what they’re* [i.e., patients] *saying…If they haven’t gone through it you might learn something from it.* [Participant 6]

Importantly, participants described how patient expertise in their lived experience of substance use can be a critical educational resource for providers, thereby improving care interactions for other patients. In these instances, participants recommended social workers and providers being trained directly by people with lived or living experience of substance use:

*Maybe just have them train or talk to them or even just sit them down with people who have lived through it who are now in recovery, you know? And just talk. Cause you know, it’s just that it’s easier to know when you’ve lived it. So if you can just talk to them and if you just understand it’s just, like, I don’t know*... [Participant 11]

Notably, participants’ narratives elucidated how a lack of lived experience among providers contributed to their inattentiveness to the complexities of patient’s lives, particularly as it intersected with substance use. One participant explained:

*Not all of us want to be addicts. Some of us want to leave this shit alone, but then again a lot of us, maybe 90% of us, are afraid of how we’re going to be treated once we get* here [the ED]*. It just really sucks. Maybe they* [physicians] *should do a little class under the bridge somewhere. Exactly, where they can see what it’s like. Not all of us want to be here.* [Participant 1]

For participants, expanding how and from whom providers learn about substance use was seen as necessary to address ongoing discrimination and stigma they regularly experienced in ED care spaces.

### “It’s kind of really up to the person”: ED-based resource utilization

While participants’ ED experiences were often shaped by whether providers and staff had lived experience, perspectives on services and resources being offered within these settings varied across participants. Notably, most participants described how they were uncertain whether immediately post-overdose was an ‘ideal time’ to recommend services or interventions to patients. This was often linked with their prior experiences of having overdosed and continuing to feel “*fogged up*” or “*disoriented*” while in the ED: “*I’ve been unfortunately in a hospital like due to overdose quite a few times in my life…I’ve just remembered always waking up so uncomfortable and disoriented*” (Participant 18). Similarly, other participants were uncertain what services, if any, they were offered while in the ED due to limited recollection of the ED visit. One participant who had been randomized to a peer recovery specialist described being uncertain as to how the interaction went:

*I don’t remember, that’s the problem. So I can’t really say. And they had no way of telling whether I was going to remember or whether I was, you know, all together there. …And I might have put on a façade that I was, I don’t know*. [Participant 5]

Given these challenges immediately following an overdose, Participant 5 continued explaining the importance of waiting until patient’s are “*in a good frame of mind*” before offering services:

*I would say at least wait a day, maybe two. …I feel if that was done in the beginning that might have been a little bit too early. Because I don’t even remember going in there* [the ED]*, so that could have been in the period where my head was all fogged up*.

Despite questioning the utility of offering referrals and services immediately following an overdose, participant narratives also drew attention to such options should be adaptive to patients rather than drawing on a one-size-fits-all approach. For many, this meant not only offering treatment or recovery options, but offering numerous supports and services that address health and ancillary needs. For example, one participant explained how referrals should be expansive:

*I think just like a range of programs cause you never know where a person’s at. Maybe someone doesn’t have a good living situation and is like really at very low might really benefit from a 28-day program…Then like another person who maybe is higher functioning like has a job and their own apartment…might really benefit from outpatient services. So I think just like a range of services for a person to pick from what’s right for them*. [Participant 18]

For many participants, offering numerous resources and service options was described as allowing participants to have more agency in decision-making about their health at a given time, rather than being “*too pushy or in* [their] *face*” about services. One participant explained:

*Case by case. I mean situations are different. I don’t feel I needed as much care with my overdose as say another person might, you know? They did ask me my situation and this and that and they were great with that. And at that time I did have a place to go…but if they want help, they’ll ask for it. …I think make them aware* [of options] *but I wouldn’t put them on it, they’ve gotta be asked.* […] *I think you should lay out all their options for them, not just recovery houses*. [Participant 14]

Importantly, participant narratives drew attention to the ways in which substance use—and the needs of people who use drugs—are heterogenous and therefore require multiple options when supporting patients.

### Improving service utilization

In addition to being offered service options and referrals, several participants described how additional supports in and outside of EDs could aid patients in service and treatment uptake decisions. Within EDs, participants described significant gaps in privacy for patients who had overdosed, as most described “*waiting out in a hallway with a bunch of other people*” while waiting to be placed in a room. The impacts of such visibility within EDs were described as potentially impacting patient utilization of services. One participant described:

*They should be put in a room for their privacy. …I think they should be put in a room where other people can’t see what they’re getting, you know what I mean? Cause maybe some people don’t wanna you know, want the other person to know their business*. [Participant 7]

Similarly, others described how establishing protocols and policies to improve patient privacy would be beneficial during patient encounters: “*Maybe there could be some sort of protocol where the person is moved to a private, more private location, to speak with a peer recovery coach*” (Participant 18).

Because participants often described not fully remembering their ED interactions following an overdose, many also stressed the importance of ensuring patients are provided with comprehensive resource and service lists prior to discharge. Such lists were positioned as allowing patients to learn about community-based resources they could access if desired and could improve engagement by offering step-by-step approaches for engaging with these services. One participant explained:

*Look up where the resources are at, get a printout of the places, then names, the phone numbers and present them* [i.e., patients] *that. Or if the person* [peer recovery specialist] *who went through it, you know, tell the patient what they did and how they did it. And if it was like one of these places, recommend something for them. …You can’t force somebody to do something they don’t want to do. The only thing you can do is put it in front of their face then they can go from there*. [Participant 6]

In addition to improving dissemination of patients’ service options, participants also shared that hospitals should provide transportation for patients following ED visits. For many, transportation limitations created barriers to leaving the ED itself, but also to accessing treatment services (e.g., outpatient treatment services). One participant described the transportation challenges they faced once discharged from the ED:

*I was still like out of it. I was real tired. And like they didn’t even have like transportation for me neither. …If I was like, you know, make a phone call, call somebody to give you a ride home. But it was like, I had to walk from the hospital downtown and I seen somebody I know and he gave me the bus fare to get back home. That should change too, you know? …From there, if a person wants the help, be able to take them from the hospital to a detox.* [Participant 4]

Here, participants drew attention to the ways in which their level of structural vulnerability intersected with health and ancillary service access in ways that could undermine post-overdose service uptake within the community.

## Discussion

This analysis of the ED experiences of patients with OUDs or at high risk for overdose demonstrates how ED-based interactions and service provision can impact ED care engagement and service utilization. Participants underscored the varied experiences of people who use drugs when accessing EDs, and how providers should build rapport with patients prior to encouraging treatment services. Specifically, participants stressed the importance of lived experience in care provision and how this can improve ED experiences through increased rapport and trust. Our study adds to a growing body of literature demonstrating the integration of peer-based supports in an ED-based setting for patients who use drugs.

Our findings document that rapport-building between some patients and providers can impact clinical experiences. In our study, rapport building was juxtaposed with ‘pushing’ services onto patients and was thus framed as an important element for reaffirming patients’ agency in ED settings. Previous research has underscored how rapport building in ED settings is important for establishing trust in care pathways and assuring patients their utilization of services or resources offered is voluntary [28, 55]. Our findings echoed this research, as participants stressed the importance of being offered services rather than being told they would receive them.

Prior research has documented how people who have an OUD or have had an overdose experience discrimination and stigma within clinical settings (e.g., [5, 31, 33, 37]). Our study reiterates these findings with participant narratives documenting the role of physical appearance, behaviors, or OUD diagnosis at adversely shaping clinical care interactions in the ED. Similar to existing research [21, 34, 46, 55], participants in our study were aware that discrimination based on their substance use was likely, and found ways to reassert their agency (e.g., disclosing their OUD diagnosis, withholding information) within clinical interactions. Recent research has assessed a range of specific interventions (e.g., narratives, visuals) to reduce stigma among healthcare providers [25]. Applying similar tactics to our study setting—and hospitals more broadly—may help reduce provider stigma related to substance use and patients who use drugs.

Peer-based supports have been well-established in community harm reduction (e.g., [24, 34, 38, 56]), treatment (e.g., [36, 50, 51]), and recovery services (e.g., [22, 29]). While research has also shown the utility of peer services in these settings at improving service engagement and health and social outcomes (e.g., reduced hospitalization, adherence to treatment) [3, 15, 30, 40], integration of peer supports within hospital settings is relatively new [28, 30, 55, 57]. Although a recent study showed that a peer-based behavioral intervention was no different than a standard intervention by clinical social work staff in an ED setting [4], our findings suggest that peer-delivered services may be beneficial for some participants due to shared experiences. Notably, the peer-delivered intervention in our parent study included three-month follow-up with patients after discharge. Future research should explore how peer-based interventions, including the impacts of prolonged engagement after discharge, inform post-overdose service utilization in and outside of ED settings.

Although the ED has been framed as an opportunistic place to provide substance use-related interventions [2, 9], participants did not always perceive the ED—and immediately post-overdose—as effective. This has been reported elsewhere [19] and highlights an opportunity to modify service and resource delivery in ED settings to better meet patients’ needs and improve engagement. Feeling confused, unable to focus, and managing stress following an overdose were described by participants as undermining their ability to engage with services being offered in the ED. Given these barriers, there may be a benefit to follow-up protocols and contact for patients discharged from the ED following an overdose as evidenced by previous research demonstrating the effectiveness of patient follow-up on improved service engagement [57]. Further, offering a range of clinical and ancillary supports (e.g., transportation supports, housing referrals, food programs) during ED visits and discharge could facilitate service engagement among some patients with varying levels of structural vulnerability.

This study has several limitations. First, the diversity of the participant sample was limited. We also were unable to reach all participants who had agreed to be contacted for the qualitative sub-study. This may have led to an underreporting of barriers and facilitators in ED settings, particularly for structurally vulnerable populations, and how this intersects across social identities. Utilizing alternative ways to reach participants (e.g., social media outreach, leaving messages with outreach workers) may be helpful for future studies. Our findings are also specific to hospitals in Rhode Island and, while they generate insights that may be relevant across other settings, they may be limited given availability of peer recovery specialists and social workers in other hospital settings. Lastly, we did not interview peer recovery specialists regarding care for patients following an overdose. Further research into their perspectives should be undertaken.

## Conclusion

In conclusion, there is an ongoing need to improve post-overdose interventions and care in the ED to reduce barriers to uptake. Modifications to post-overdose care should be informed by patient experiences and include supports that address social and structural needs across patient populations.

## Data Availability

The datasets generated and analyzed during this study are not publicly available as this would violate the agreement to which the participants consented, but are available from the corresponding author upon reasonable request.
